# AP2: an indispensable host factor in virus infection

**DOI:** 10.1128/jvi.02164-25

**Published:** 2026-01-21

**Authors:** Rui Li, Yan Jiang, Xinrong Wang, Longxiang Zhang, Yue Wang

**Affiliations:** 1 College of Veterinary Medicine, Southwest University 26463, Chongqing, China; 2 National Center of Technology Innovation for Pigs, Chongqing, China; New York University Department of Microbiology, New York, New York, USA

**Keywords:** adaptor protein complex 2, virus cellular transport, virus life cycle, antiviral drug targets, immune evasion

## Abstract

Adaptor protein complex 2 (AP2), a central regulator of clathrin-mediated endocytosis and intracellular cargo trafficking, is hijacked by numerous viruses to complete their infectious cycles. This review systematically synthesizes the multifaceted roles of AP2 across the entire viral life cycle, from entry and replication to assembly and release, as well as in immune evasion. By delineating how diverse viruses exploit this key host machinery, we further consolidate the rationale and current progress in developing broad-spectrum antiviral strategies that target AP2 and its regulatory pathways. This work aims to provide a unified perspective on AP2 as a critical host-pathogen interface, offering new insights into viral pathogenesis and antiviral drug discovery.

## INTRODUCTION

Viral transport within host cells is critical for viral replication. The dense cytoskeletal network and specialized organelles in the cytoplasm create a complex microenvironment that severely restricts free diffusion of molecules exceeding 500 kDa (1, 2). Given their substantial size, often spanning tens to hundreds of nanometers in diameter, viruses cannot diffuse freely through the cytoplasm. Consequently, they must hijack host transport systems to reach their replication sites efficiently. The viral infection process typically involves several key steps: (i) attachment to host cell surface receptors, (ii) entry through the clathrin-mediated endocytosis (CME) pathway or membrane fusion, (iii) genome release, (iv) intracellular transport of viral genetic material to replication sites, (v) coordinated trafficking of viral components for assembly, and (vi) progeny virus release. Adaptor protein complexes (APs), essential regulators of transmembrane trafficking, are hijacked by viruses to mediate intracellular transport at multiple stages of infection.

APs are heterotetrameric adaptor proteins that mediate the sorting of cargo proteins to specific intracellular membranes (3). Five APs have been identified in mammalian cells, designated as AP1, AP2, AP3, AP4, and AP5 (4), of which AP2 is the most extensively studied (5). As a key initiator of CME, AP2 recruits clathrin to the plasma membrane to drive coat assembly and cargo uptake, ensuring the orderly progression of the CME pathway. Beyond its role at the plasma membrane, AP2 also binds to diverse intracellular membranes, including the Golgi apparatus and the endoplasmic reticulum (ER), facilitating vesicle trafficking between these organelles (6). AP2 plays a critical role in viral infections through multiple mechanisms: (i) mediating viral internalization via the CME pathway, (ii) redirecting viral protein localization to promote replication, assembly, and release, and (iv) modulating host immune evasion and cellular functions (Table 1).

**TABLE 1:** Viral families and their interactions with AP2 subunits including the stages at which they act^*a*^

Group	Viral families and members		AP2-associated viral proteins or cellular proteins	AP2 subunits involved	Function
DNA virus	*Adenoviridae*	AdV/human adenovirus D37 (HAdV-D37)	RIDα, RIDβ/epidermal growth factor receptor (EGFR), Fas, TRAIL-R1, and TRAIL-R2	AP2M1	Immune evasion (7–11)
			−/−	AP2A1	Internalization (12)
	*Papillomaviridae*	Bovine papillomavirus (BPV)	−/−	–	Internalization (13)
		Human papillomavirus (HPV)	E7/EGFR, MET, and CD109	AP2M1	Immune evasion (14, 15)
			−/−	–	Internalization (13)
	*Hepadnaviridae*	Hepatitis B virus (HBV)	LHBsAg/−	AP2A1/2	Internalization (16, 17)
	*Polyomaviridae*	JC polyomavirus (JCPyV)	−/5-HT_2_Rs, β-arrestin	AP2B1	Internalization (18)
	*Parvoviridae*	H-1 parvovirus (H-1PV)	−/−	AP2M1	Internalization (19)
	*Herpesviridae*	Epstein-Barr virus (EBV)	Viral GPCR (vGPCR) (BILF1)/−	AP2M1	Immune evasion (20)
		Porcine lymphotropic herpesvirus (PLHV)	vGPCR (BILF1)/−	AP2M1	Immune evasion (20)
		KSHV	−/EphA2 and Esp15	AP2A1/2/AP2S1	Internalization (21, 22)
			vGPCR/−	AP2M1	Immune evasion (23)
		Pseudorabies virus (PRV)	gB/−	AP2M1	Release (24)
					Immune evasion (24)
			−/−	AP2M1	Internalization (25)
			gE/−	AP2M1?	Release (26)
		Murine cytomegalovirus (MCMV)	m04/MHC-1	AP2M1	Immune evasion (27)
	*Poxviridae*	Vaccinia virus	F13L and B5/−	AP2M1/AP2S1	Assembly (28–30)
			A36/intersectin-1 and Eps15	AP2A1/2/AP2B1	Release (31)
	*Nimaviridae*	White spot syndrome virus (WSSV)	VP37 and VP26/−	AP2B1/AP2S1	Internalization (32–34)
RNA virus	*Retroviridae*	Equine infectious anemia virus (EIAV)	Gag(p9)/−	AP2M1	Assembly (35)
			Gag(p9)/−	AP2M1	Release (36)
			S2/SERINC5 and SERINC3	AP2S1	Immune evasion (37)
		HIV-1	Env/−	AP2M1/AP2S1	Assembly (38, 39)
			TM/−	AP2B1	Immune evasion (40)
			Gag/−	AP2M1	Assembly (41, 42)
					Release (41, 42)
			Nef/V1H, Tetherin, CD28, CD8αβ, CD3, CD4, SERINC3, SERINC5, MHC-1, and MHC-II	AP2M1/AP2S1	Immune evasion (43–55)
			Vpu/Tetherin, Serinc3, and CD4	AP2M1/AP2S1	Immune evasion (56–60)
					Release (61)
			−/−	AP2A1/2	Replication (62)
		HIV-2			Assembly (63)
					Release (61)
					Immune evasion (64)
			Nef/CD4, CD28, CD8αβ, and MHC-1	AP2S1	Immune evasion (65, 66)
		Murine leukemia virus (MLV)	Env/−	AP2M1	Release (61)
			glycoGag/−	AP2M1	Release (67)
					Immune evasion (68, 69)
		Human T cell leukemia virus type 1 (HTLV-1)	Env/−	AP2M1	Assembly (70)
					Release (71)
		Simian immunodeficiency virus (SIV)	Nef/V1H, Tetherin, CD3, CD28, and CD4	AP2M1/AP2S1/AP2B1	Immune evasion (43, 45, 50, 72–82)
			TM/−	AP2B1	Immune evasion (40)
			Env/−	AP2M1	Assembly (38, 72, 83)
					Release (38, 72, 83)
	*Filoviridae*	Ebola virus	GP/−	AP2A1/2	Internalization (84)
			−/−	AP2M1	Assembly (85)
	*Coronaviridae*	Human coronavirus 229E (HCoV-229E)	−/−	AP2M1	Internalization (86)
		Middle East respiratory syndrome coronavirus (MERS-CoV)	Nucleoprotein (NP)/−	AP2M1	Replication (87)
		Severe acute respiratory syndrome coronavirus-2 (SARS-CoV-2)	−/Angiotensin-converting enzyme 2 (ACE2)	AP2M1	Internalization (88, 89)
			−/−	AP2A2 (90)	-
		Severe acute respiratory syndrome coronavirus-1 (SARS-CoV-1)	−/ACE2	AP2M1	Internalization (89)
	*Flaviviridae*	Hepatitis C virus (HCV)	−/−	AP2M1	Internalization (91–93)
			core/−	AP2M1	Replication (94)
			core/EGFR	AP2M1	Assembly (5, 93–95)
		Zika virus (ZIKV)	NS3/−	AP2M1	Replication (87)
			−/−	AP2B1	Internalization (96)
		Dengue virus (DENV)	−/−	AP2M1	Internalization (85, 97)
			−/−		Release (85, 97, 98)
	*Picornaviridae*	Enterovirus A71 (EV-A71)	−/−	AP2A1	Internalization (99)
			2C/−	AP2M1	Replication (87)
		Human rhinovirus (HRV)	−/−	−/−	Internalization (100, 101)
	*Orthomyxoviridae*	IAV	NP/−	AP2M1/AP2S1	Replication (87, 102)
			M1 and NA/−	AP2S1(190)	–
			−/Free fatty acid receptor 2 (FFAR2) and β-arrestin1	AP2B1	Internalization (103)
	*Paramyxoviridae*	Newcastle disease virus (NDV)	−/−	AP2A2 (104)	–
			F/−	AP2M1	Release (105)
	*Rhabdoviridae*	Rabies virus (RABV)	−/−	AP2M1	Internalization (106)
		Vesicular stomatitis virus (VSV)	−/−	AP2A1/2	Internalization (107)
	*Bunyavirales*	Crimean-Congo hemorrhagic fever virus (CCHFV)	−/−	–	Internalization (108)
		Toscana virus (TOSV)	−/−	AP2M1	Internalization (109)

^*a*^ – indicates that the item has not yet been identified.

While the canonical role of AP2 in the CME pathway is well-characterized and previous reviews have covered the AP family broadly or viral entry mechanisms, a systematic synthesis of AP2’s multifaceted involvement across the entire viral life cycle is notably lacking. This review aims to fill this critical gap by presenting a unified perspective that delineates how diverse viruses exploit AP2 during entry, intracellular trafficking, replication, assembly, release, and immune evasion. Furthermore, we uniquely consolidate and discuss the emerging potential of AP2 and its regulatory kinases as targets for broad-spectrum antiviral strategies. By framing AP2 as a central hub in the host-virus interface, we provide a comprehensive resource that underscores the strategic potential of targeting host trafficking machinery to combat a wide range of viral threats.

## AP2 PHYSIOLOGICAL FUNCTIONS

### Composition of AP2 and functions of its subunits

AP2 is a heterotetrameric complex composed of four subunits: α, β, μ, and σ. The α subunit has two isoforms: AP2-αA (AP2A1) and AP2-αB (AP2A2), encoded by the genes *AP2A1* and *AP2A2*, respectively (110). This subunit binds to phosphatidylinositol-4,5-bisphosphate (PIP_2_) at the plasma membrane to help recruit AP2 (91), and together with the σ subunit (AP2S1), recognizes the [E/D]XXXL[L/I] (whereas X refers to any amino acid) sorting motif in cargo proteins (111). The β subunit (AP2B1) serves as a critical link between AP2 and clathrin during vesicle formation. It also contributes to the selective recruitment of cargo and facilitates microtubule-dependent trafficking by interacting with dynein, ensuring proper subcellular localization (112). Additionally, AP2B1 may participate in regulating specific cargo selection during membrane transport processes (113). The μ subunit (AP2M1) specifically interacts with the YXXΦ sorting motif (Φ indicates hydrophobic residues including L/M/F/I/V) in the cytoplasmic tails of cargo proteins, thereby enabling cargo recognition and sorting (87).

### Assembly of AP2

**Fig 1:**
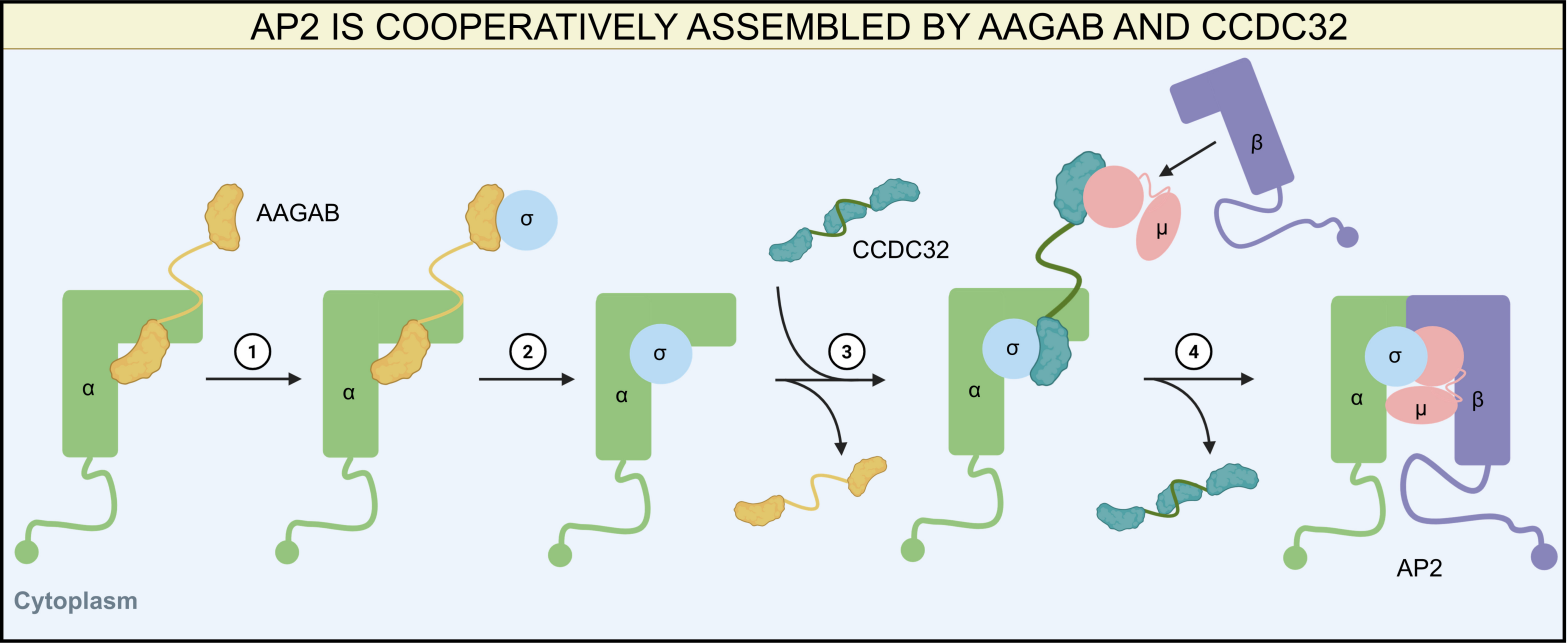
Assembly of the AP2 heterotetramer. ① AAGAB first binds the α/σ subunit to generate an α-AAGAB-σ ternary complex, and then ② AAGAB dissociates from the heterotrimer: ③ CCDC32 displaces AAGAB and recruits the μ/β subunit to form a CCDC32-α-σ-μ-β pentameric intermediate, and ④ CCDC32 dissociates from the heteropentamer to form a functional AP2 complex.

Research on the assembly of AP2 into a heterotetramer began relatively late (114) and has revealed a chaperone-dependent process involving AAGAB and CCDC32. AP2 assembly is initiated by the binding of the C-terminal domain (CTD) of AAGAB to the α subunit, forming an AAGAB-α binary complex (115). Subsequently, AAGAB binds the σ subunit via its N-terminal pseudo-GTPase domain (116), generating an α-AAGAB-σ ternary complex (117). CCDC32 then recognizes this ternary intermediate and interacts directly with both the α and σ subunits through multiple interfaces (118). The association of CCDC32 first forms a CCDC32-α-AAGAB-σ quaternary complex, after which the binding of CCDC32 triggers the release of AAGAB, resulting in a stable CCDC32-α-σ ternary complex (119). Within CCDC32, a WXXΦ motif can mimic the canonical YXXΦ motif and bind to the CTD of the μ subunit (118), thereby recruiting the μ subunit to generate a CCDC32-α-σ-μ quaternary complex. Next, either the β subunit alone or a pre-formed μ-β sub-complex is recruited, leading to a CCDC32-α-σ-μ-β pentameric intermediate. Upon completion of the heterotetramer assembly, CCDC32 dissociates, leaving a fully functional AP2 complex (Fig. 1).

### Post-translational modifications of AP2

Post-translational modifications of AP2 primarily involve phosphorylation, although ubiquitination and other modifications have also been reported. Among the subunits, AP2A1/2, AP2B1, and AP2M1 are known to undergo phosphorylation (120), while no phosphorylation sites have been identified on AP2S1 (121). AP2B1 is phosphorylated in the cytoplasm, but this modification is reversed upon AP2 binding to the plasma membrane. Failure to dephosphorylate AP2B1 impairs its interaction with clathrin, impairing vesicle formation (122).

**Fig 2:**
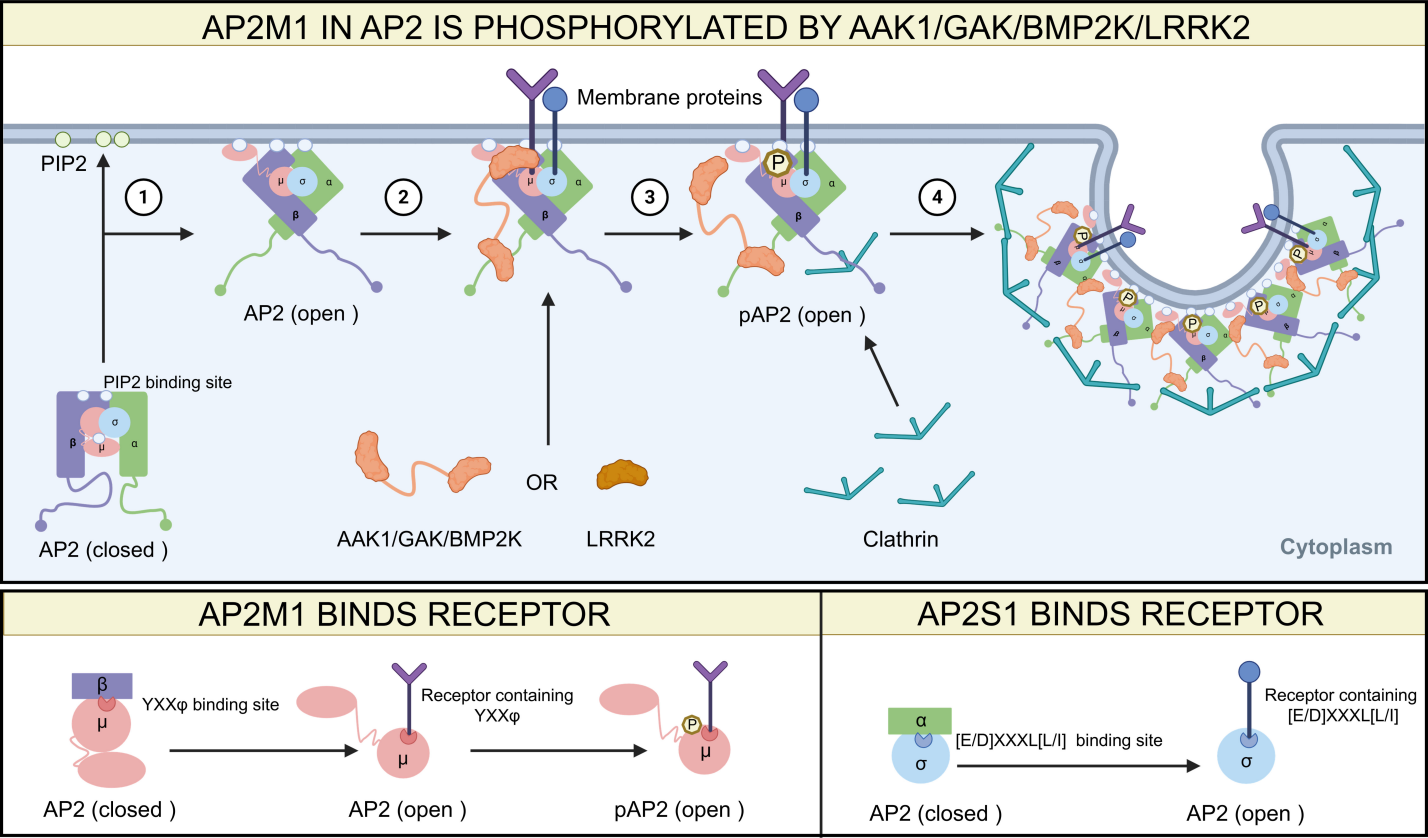
Structural schematic and phosphorylation modification of AP2. Top panel: schematic diagram of conformational changes of AP2. ① AP2 subunits αA/B (AP2A1/2), β (AP2B1), and μ (AP2M1) each contain PIP2-binding sites. AP2 is recruited to the cell membrane, where its subunits αA/B, β, and μ bind to PIP2, switching from a closed to an open conformation. ② AP2 with an open conformation exposes its cargo-binding pockets on μ and σ (AP2S1), thereby allowing the membrane proteins to bind. ③ AAK1/GAK binds to αA/B, BMP2K binds to αA/B or β, and LRRK2 binds to μ, ultimately stabilizing cargo binding by phosphorylation of the T156 residue on μ. ④ After phosphorylation of subunit μ, the AAK1, GAK, and BMP2K remain temporarily associated with AP2, serving as scaffolds to support CCP formation and maturation. Bottom panel: details of the AP2-binding sites. In the closed conformation of AP2, the clathrin-binding site on β is buried, the YXXφ motif-binding site on μ is masked by β, and the [E/D]XXXL[L/I] motif-binding site on σ is enshrouded by αA/B. Upon the conformation of AP2 transitions to an open state, the phosphorylation site at T156 residue on μ becomes exposed.

Current research primarily focuses on the phosphorylation of AP2M1 at the threonine 156 (T156) (123), and this modification is mediated by several kinases, including adaptor-associated kinase 1 (AAK1) (124), cyclin G-associated kinase (GAK) (125), BMP-2-inducible kinase (BIKE/BMP2K) (126), and leucine-rich repeat kinase 2 (LRRK2) (127, 128). Phosphorylation of AP2M1 at T156 is crucial for its activity, as loss of this modification disrupts cargo binding and inhibits endocytosis (124). AAK1 and GAK phosphorylate AP2M1 by binding to AP2A1/2, while BMP2K may bind to AP2A1/2 or AP2B1. In contrast, LRRK2 directly binds to and phosphorylates AP2M1. Following phosphorylation of AP2M1 at T156, these kinases can remain associated with the AP2 to participate in subsequent stages of CME. Phosphorylation of AP2M1 at T156 enhances the recruitment of AP2 to the plasma membrane (129) and stabilizes its affinity for tyrosine-based sorting signals in cargo proteins, thereby promoting cargo internalization (124), regulating clathrin-coated pit (CCP) maturation, and influencing the rate of CME (130). This modification may also induce a conformational shift in AP2M1 from a closed to an open state, exposing the YXXΦ motif-binding site of AP2M1 to facilitate cargo recognition and engagement (121) (Fig. 2).

Rinaldi et al. (131) have identified ubiquitination sites on AP2M1. The E3 ubiquitin ligase Praja2 was shown to recruit and ubiquitinate AP2M1, enabling the modified protein to bind membrane-associated proteins and facilitate their degradation. However, it remains unclear whether Praja2-mediated ubiquitination regulates AP2 assembly, stability, or interactions with proteins involved in the early stage of endocytic vesicle formation and intracellular trafficking. Further investigation is needed to elucidate these potential regulatory mechanisms.

### Physiological functions of AP2

#### AP2 in transmembrane and cytosolic cargo trafficking

AP2 recognizes specific endocytic motifs, within the cytoplasmic tails of transmembrane proteins. This process governs the internalization of critical signaling molecules like vascular endothelial growth factor receptor 2 (VEGFR2) (132), MHC-related antigen 1 (133), and sodium/iodide symporter (134). Proper AP2 function is essential for maintaining cellular homeostasis, and its dysfunction is linked to mislocalization of these cargoes, contributing to the development and progression of various cancers (131, 135–137). Beyond transmembrane proteins, AP2 also coordinates the trafficking of cytosolic cargo through interactions with specialized adaptors. For example, AP2B1 forms a complex with BubR1 and MAD2 to recruit the insulin receptor into clathrin-coated vesicles (CCVs), thereby promoting signal transduction (138, 139).

Furthermore, AP2 can serve as an intermediary molecule between the CME pathway and lipid raft. AP2 can recognize the lipid raft-localized protein Tetherin (CD317/BST-2) through its AP2M1-binding motif in its cytosolic tail, followed by internalization through the CME pathway. In this process, the lipid raft localization of Tetherin is crucial for its AP2-dependent internalization process (140). Consistent with this role, AP2 also participates in controlling the retrieval and endocytic traffic of cytolytic machinery components. Following NK cell activation, Munc13-4 becomes enriched in lipid rafts, where AP2 is recruited via its interaction with PIP2 or PIP5Kγ. AP2 then likely recognizes a sorting motif in the cytosolic tail of Munc13-4 to mediate its endocytic trafficking (141, 142).

This fundamental role of AP2 in cellular trafficking makes it a frequent target of viral exploitation. Many viruses have evolved to mimic host cargo motifs, thereby effectively hijacking the AP2 transport machinery to facilitate viral entry, intracellular trafficking, replication, and immune evasion, as detailed in the following sections.

#### AP2 in nervous system signaling

AP2 plays a critical role in regulating synaptic vesicle formation and release (143). Not only is it essential for CME, ultrafast endocytosis, and the dynamin-dependent scission of coated vesicles at most synapses across model organisms (144, 145), but it also facilitates these processes by recruiting clathrin, assembling endocytic machinery, and concentrating cargo proteins at sites of vesicle formation. It has been proposed that AP2 also helps translocate SV proteins from the active zone to the periactive area, where endocytosis occurs (143). Dysfunction of AP2-dependent trafficking disrupts neuronal signaling pathways and contributes to several neurological disorders, including Parkinson’s disease (127, 128), epilepsy (146, 147), Alzheimer’s disease (148–152), and others (153, 154). Notably, inhibition of AP2 has been shown to suppress pain responses (155), a vulnerability that neurotropic viruses may exploit to enhance their release from host neurons.

#### AP2 in cellular signaling and pathogenesis

AP2 modulates several critical signaling pathways, including the mTOR, Wnt/β-catenin, and NF-κB signaling pathway, to regulate key cellular processes such as proliferation (156), differentiation (157), morphology (158), apoptosis (159–161), and autophagy (162–166). In the mTOR and Wnt/β-catenin signaling pathways, AP2 serves as a critical node integrating the mTOR and Wnt/β-catenin pathways (167). It activates mTORC1 (156), which in turn promotes the interaction of DVL and AP2 to enhance FZD internalization (157). Accordingly, silencing of AP2B1 inhibits mTOR signaling and reduces the surface expression of the α-amino-3-hydroxy-5-methyl-4-isoxazolepropionic acid receptor subunit GluA2, an effect rescued by restoring mTOR/S6K1 activity or overexpressing GluA2 (158), although the precise mechanisms remain to be elucidated.

AP2 also influences the NF-κB signaling pathway. It has been reported to modulate IκB-α degradation and TNF-α release independently of its canonical trafficking functions (168). Furthermore, AP2 contributes to NF-κB pathway activation by transporting membrane-localized activators such as CXCR2 and TLR9 (169, 170). Dysregulation of AP2 has also been linked to skin disorders, such as punctate palmoplantar keratoderma type I (171) and hyperpigmentation (172).

**Fig 3:**
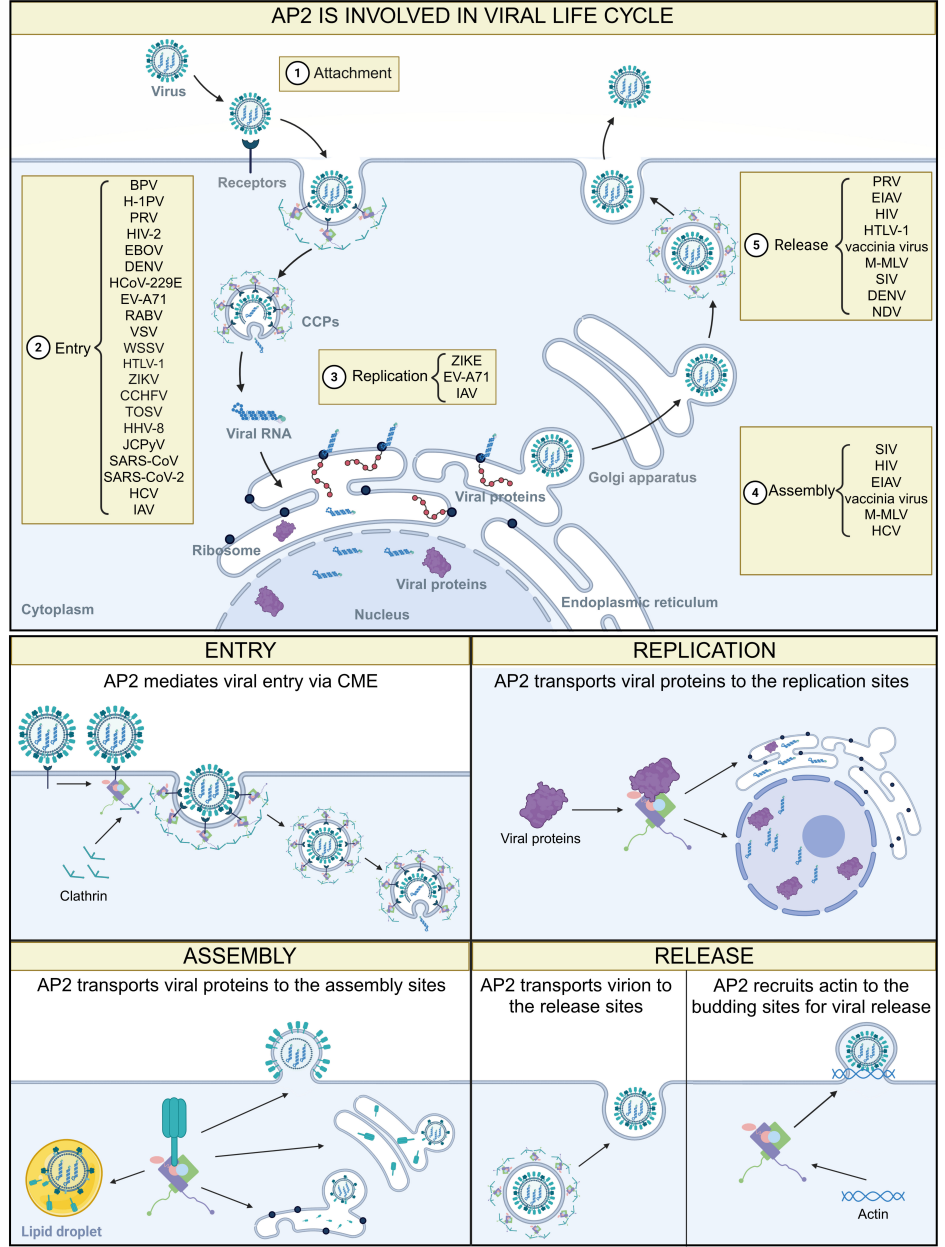
AP2 is involved in multiple stages of the viral life cycle. Top panel: overview diagram of AP2 involvement in the various stages of the viral life cycle. ① AP2 is not involved in viral attachment. ② AP2 mediates viral entry. ③ AP2 supports viral replication. ④ AP2 contributes to viral assembly. ⑤ AP2 participates in viral release. Bottom panel: schematic of the detailed mechanisms of AP2 involved in the viral life cycle. AP2 mediates viral entry from the cell membrane to the cytoplasm via the CME pathway (top left row). AP2 transports viral replication-associated proteins to the ER or nucleus for genome replication, transcription, and translation (top right row). AP2 transports viral components to lipid droplets, ER, Golgi apparatus, or plasma membrane for virion assembly (bottom left row). AP2 transports the mature virions to plasma membrane for release or recruits actin to promote the scission of virus-containing vesicles from the budding sites (bottom left row).

Importantly, the multifaceted regulatory roles of AP2 in cellular signaling represent a point of vulnerability that is frequently exploited by viruses. Through hijacking AP2-mediated trafficking and signaling pathways, viruses can actively reprogram host cell fate, alter cellular morphology, manipulate apoptotic responses, and reshape metabolic states, thereby creating favorable conditions conducive to viral entry, replication, and spread (Fig. 3). This convergence of AP2-dependent signal regulation and trafficking highlights AP2 as a strategically important host factor at the interface of normal cellular physiology and viral pathogenesis.

## ROLE OF AP2 IN VIRAL INFECTION

### Role of AP2 in early viral infection

#### AP2 assists in viral invasion via the CME pathway

AP2 plays a central role in viral entry through the CME pathway, which is utilized by the majority of viruses following attachment to host cell receptors. This process relies heavily on AP2’s ability to coordinate the formation of CCVs, which encapsulate viral particles for internalization. Numerous viruses, including BPV (13), JCPyV (18), H-1PV (19), PRV (25), human immunodeficiency virus-2 (HIV-2) (64), Ebola virus (84), DENV (85, 97), HCoV-229E (86), EV-A71 (99), RABV (106), CCHFV (108), and VSV (107), have been observed within AP2/CCVs. Disruption of AP2 function severely impairs the ability of these viruses to enter host cells, underscoring its critical role in this process. Nevertheless, the precise molecular mechanisms linking AP2 to viral receptors remain an active area of investigation.

The CME-dependent viral entry mechanism involves three key stages. First, viruses bind to specific receptors or co-receptors on the host cell surface. This interaction triggers conformational changes in the transmembrane receptors or co-receptors to expose endocytic motifs, which recruit AP2. AP2 then initiates the assembly of clathrin, leading to the formation of CCVs that encapsulate the viral cargo. During vesicle maturation, AP2 acts as a central hub, coordinating the interaction among viral components, membrane lipids, clathrin, and various accessory proteins to ensure proper CCV formation and membrane scission (91). For instance, the WSSV exploits this pathway by utilizing its VP24 protein to bind the host receptor MjpIgR (polymeric immunoglobulin receptor-like protein), thereby hijacking the AP2-mediated CME pathway for cellular entry (32).

Both SARS-CoV-1 and SARS-CoV-2 exploit ACE2 as a receptor for host cell attachment. The cytoplasmic tail of ACE2 contains a conserved YXXΦ motif that can bind AP2M1 (88). Notably, SARS-CoV-2 entry has been shown to depend on AP2 activity, independent of ACE2’s downstream signaling function, which suggests the presence of other accessory factors that facilitate SARS-CoV-2 internalization (89). HCV, potentially through a mechanism similar to that proposed for SARS-CoV, may exploit the YXXΦ motif within the cytoplasmic tails of receptors such as CD81, CD63, CD82, CD151, and EGFR to recruit AP2, thereby facilitating viral entry via the CME pathway (91–93). By integrating viral components into the host endocytic machinery, AP2 not only enables viral entry but also indirectly supports immune evasion and intracellular trafficking. The reliance of diverse viruses on this pathway highlights AP2 as a critical host factor and a promising target for antiviral therapeutics. Further studies are needed to unravel the dynamic interplay between AP2, viral receptors, and co-opted host factors during infection.

AP2 can mediate viral invasion indirectly by interacting with bridging factors, which recruit it to the plasma membrane and link it to key viral receptors. This forms a receptor-bridging factor-AP2 assembly that triggers the CME pathway. For instance, JCPyV binds to 5-HT2Rs, activating intracellular signaling pathways that mobilize the bridging factor β-arrestin. Activated β-arrestin subsequently recruits AP2 to form a 5-HT2R-β-arrestin-AP2 complex, enabling CME-dependent viral entry (18). Similarly, the avian influenza virus (AIV) engages FFAR2, which interacts with β-arrestin1 to assemble an FFAR2-β-arrestin1-AP2B1 complex, facilitating viral internalization (103). In contrast, HRV exploits the low-density lipoprotein receptor (LDLR), which contains a YXXΦ motif (100, 101) but primarily relies on the bridging factor Disabled-2 (Dab2) to link LDLR with AP2, forming an LDLR-Dab2-AP2 complex that facilitates CME-mediated entry (173). These examples illustrate how diverse viruses co-opt bridging factors to physically tether AP2 to their receptors, hijacking the clathrin machinery for successful host cell invasion.

AP2 has been strongly implicated in mediating HBV entry into host cells via CME (16, 17). HBV entry relies on interactions between the pre-S1 domain of its large surface antigen (LHBsAg) and putative host receptors, although the precise mechanisms remain unclear. Research by Hsiu-Chen Huang’s group demonstrated that the pre-S1 domain of LHBsAg can directly bind to AP2, suggesting that AP2 may regulate HBV internalization via the CME pathway through this interaction (16). However, several critical questions remain unanswered: How does the membrane-associated LHBsAg engage with the cytosolic AP2? Does AP2 initiate the CME pathway by directly interacting with HBV receptors? Could AP2 also participate in the intracellular trafficking of LHBsAg to facilitate viral assembly or release?

#### Role of AP2 in alternative pathways of viral host invasion

AP2 has also been implicated in the viral entry pathway beyond classical CME. For example, AP2 participates in a form of noncanonical CME, which is actin-dependent and functions independently of traditional clathrin-associated components, such as epsin, endosomal acidification, or EEA1, to mediate the entry of HAdV-D37 (12). These findings highlight AP2’s role as a versatile adaptor and its potential as a convergent target for viruses using diverse entry pathways. Consequently, therapeutic strategies that disrupt AP2 function (e.g., via kinase inhibition) could hold broad-spectrum potential by targeting viruses that rely on distinct entry mechanisms.

### Role of AP2 in viral genome replication

AP2 plays a pivotal role in viral genome replication by regulating the subcellular localization of viral proteins and nucleic acids. As a key trafficking adaptor, AP2 mediates the transport of viral components by interacting with YXXΦ motifs in replication-associated proteins. For example, the NP of AIV, which serves as the scaffold for viral ribonucleoprotein complexes (vRNPs), contains a YXXΦ motif that binds AP2M1, enabling AP2-dependent nuclear import of vRNPs to ensure efficient viral replication (87). Similarly, the 2C protein of EV-A71 and the NS3 protein of ZIKV use their YXXΦ motifs to interact with AP2M1, directing them to the ER membrane, where they promote viral genome replication (174). Interestingly, knockdown of AP2A1/2 has been reported to enhance viral nucleic acid replication in certain contexts, potentially by modulating the nuclear import of viral genomes (62), although the precise mechanisms remain unclear. While AP2’s role in promoting replication is evident for viruses such as IAV, EV-A71, and ZIKV, its involvement in the replication of HIV-1 requires further exploration. These findings underscore AP2’s dual functionality as both a facilitator of viral trafficking and a potential regulatory node, with context-dependent effects on replication efficiency across diverse viral families.

### Role of AP2 in late-stage viral infection

AP2 acts as a critical mediator of intracellular trafficking for diverse viruses, facilitating viral assembly and release through two primary mechanisms. First, it directly interacts with viral proteins via its cargo-recognition domains, transporting them to specific subcellular locations, such as the plasma membrane or others viral assembly and/or release sites, to ensure proper virion assembly and release (175). Second, AP2 engages with host proteins essential for viral morphogenesis and budding, such as endocytic sorting complex required for transport and actin-regulating factors, to coordinate the late stages of infection. These interactions ensure spatial and temporal precision in viral particle maturation and egress, facilitating efficient propagation of the virus.

#### Role of AP2 in late-stage viral infection via direct interaction with viral proteins

AP2 regulates viral assembly by binding viral proteins and directing their intracellular trafficking to specific assembly sites. Viral proteins recruit AP2 to mediate the intracellular trafficking of viral proteins. For example, the HCV core protein hijacks AP2M1 via its YIPL/V motif, promoting the recruitment of AP2M1 to lipid droplets. AP2M1 then facilitates the trafficking of the core protein to the Golgi and ultimately to the ER, where HCV assembly is orchestrated (5). This exemplifies how viruses co-opt AP2’s cargo-sorting machinery to spatially coordinate virion morphogenesis.

After translation and post-translational modification, viral envelope proteins transiently localize to the plasma membrane. Meanwhile, leveraging its broad specificity for cargo recognition and quality control functions, AP2 redirects cell surface-localized proteins back to intracellular compartments, a process critical for receptor recycling (176). By exploiting this mechanism, the virus disguises its envelope proteins as host membrane proteins. Subsequently, AP2 internalizes these proteins to either concentrate viral components at specific assembly sites or redistribute scattered membrane-associated proteins to predefined locations for coordinated virion assembly and/or release. For example, the poxvirus B5R protein relies on AP2-mediated retrograde trafficking from the plasma membrane to the Golgi apparatus to facilitate viral morphogenesis (28). This “hide-and-redirect” strategy enables precise spatiotemporal control of viral assembly and release while evading immune surveillance.

#### Role of AP2 in late-stage viral infection through host protein interactions

AP2 can also be indirectly recruited to viral assembly sites through interactions with host proteins that associate with AP2, such as clathrin, GGAs, or other adaptor protein complexes. These interactions enable AP2-mediated trafficking to specific subcellular compartments. For example, the poxvirus F13L protein is transported to the plasma membrane via the secretory pathway. Although Matloob Husain (29) demonstrated a specific co-immunoprecipitation between F13L and AP2A1/2, mutations in putative interaction motifs within F13L did not disrupt this binding, suggesting an indirect interaction. One proposed mechanism involves AP2A1/2 binding to trafficking adaptors like Eps15 (177), which may serve as a bridge to connect F13L with AP2, forming an F13L-Eps15-AP2 complex that mediates retrograde transport of F13L from the plasma membrane to the Golgi apparatus for poxvirus assembly. Similarly, the poxvirus A36 protein recruits AP2 by interacting with intersectin-1 and Eps15, forming an A36-intersectin-1/Eps15-AP2 complex. In this context, AP2 facilitates the polarization of A36 and N-WASP at the plasma membrane, driving rapid and sustained actin polymerization to propel viral release (31). These examples highlight how viruses exploit AP2’s adaptor network through indirect host protein-mediated interactions to coordinate assembly and egress, although the precise molecular mechanisms require further elucidation.

#### Role of AP2 in late-stage infection of enveloped viruses

AP2 coordinates the spatiotemporal regulation of envelope protein trafficking in enveloped viruses, enabling focused virion assembly and immune-evasive budding (178). Viral proteins transported to the cell membrane subsequently hijack AP2’s cargo-sorting machinery through endocytic motifs (e.g., YXXΦ and YQRL) to cluster at specialized membrane microdomains. In HIV-1, for instance, AP2 mediates the polarized redistribution of Env (38, 39) and Gag (41, 42) proteins to lipid raft regions, where coordinated assembly and budding occur. Similarly, HTLV-1 Env (70), SIV Env (38, 72, 83), HIV-2 Env (63), EIAV p9 (35, 36), and NDV F protein exploit AP2-dependent trafficking to concentrate at viral budding sites. PRV provides a striking example of AP2’s compartmentalization precision. For example, its gB protein recruits AP2M1 via a YQRL motif, redirecting basolateral transport to enhance viral release while evading apical immune surveillance. This AP2-mediated redistribution strategy balances two competing needs: concentrating envelope proteins at budding sites ensures efficient virion incorporation during budding, while limiting overall surface exposure minimizes antibody neutralization and T cell recognition, thereby promoting persistent infection. By spatially confining viral assembly and temporally regulating envelope protein presentation, AP2 acts as a master regulator at the host-pathogen interface during late-stage enveloped virus infection.

AP2 enables enveloped viruses to fine-tune the assembly and release by spatially restricting budding events and modulating viral protein secretion. In HIV-2, the Env protein interacts with AP2M1, while the Gag protein further engages AP2M1 via its YXXΦ motif, paradoxically inhibiting premature viral release to ensure coordinated budding (41, 42). This dual interaction suggests AP2 not only clusters viral components at budding sites but also acts as a "molecular brake," preventing untimely secretion of viral proteins and instead retaining them at pre-assembly hubs. By enforcing precise subcellular localization, AP2M1 ensures high-density clustering of viral structural proteins at defined membrane domains, which promotes efficient virion morphogenesis while restricting budding to distinct microdomains (41). This strategic control over budding dynamics minimizes membrane destabilization, thereby prolonging host cell viability to sustain viral particle production. Such spatiotemporal regulation exemplifies how viruses repurpose AP2’s trafficking precision to balance rapid propagation with immune evasion, optimizing their fitness during persistent infections.

### Alternative mechanisms of AP2 in viral infection

**Fig 4:**
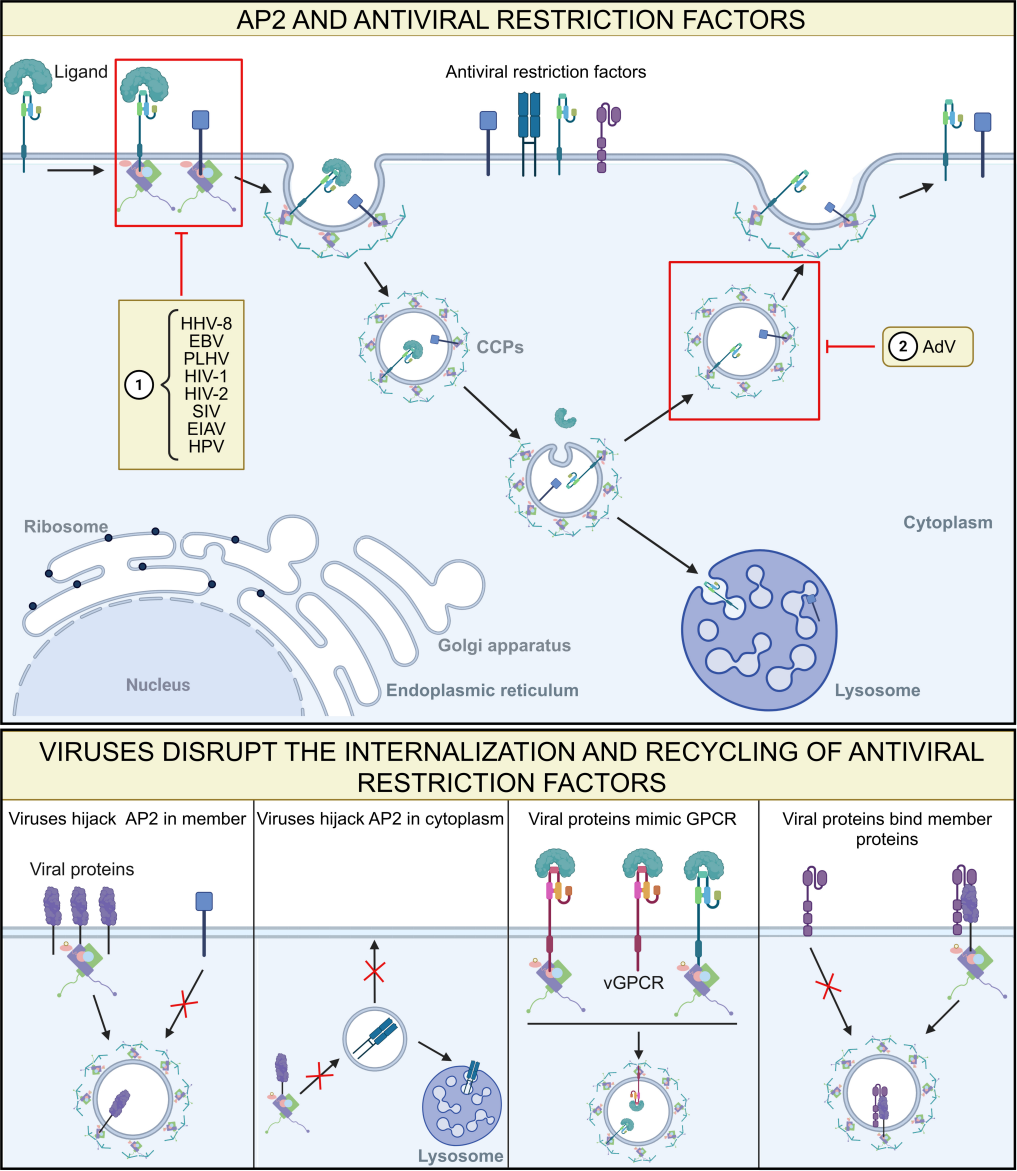
Viruses exploit AP2 to evade the host immune system. Top panel: viral interference with specific stages of antiviral restriction factors involving AP2. ① Viruses utilize AP2 to disrupt the internalization of antiviral restriction factors into the cytoplasm. ② Viruses exploit AP2 to interfere with the recycling of restriction factors to the cell membrane. Bottom panel: detailed mechanisms by which viruses exploit AP2 to interfere with restriction factors. Viral membrane-associated proteins sequester AP2 near the plasma membrane and inhibit the trafficking of immune restriction molecules (first column). Viral proteins sequester AP2 in the cytoplasm and prevent its correct localization at the plasma membrane by trapping immune restriction factors within vesicles or delivering them to lysosomes for degradation (second column). Viral proteins mimic the AP2-binding proteins (e.g., GPCR) to participate in signal transduction during infection (third column). Viral proteins bind to AP2 within cells, impairing the recycling of the immune restriction molecules (fourth column).

Beyond its role in viral entry, AP2 has been implicated in reshaping host cellular physiology during infection through multiple mechanisms, including the redistribution of host proteins and the modulation of immune receptor surface expression (Fig. 4). These coordinated actions not only facilitate viral immune evasion but also disrupt cellular homeostasis, leading to aberrant signaling and metabolic dysregulation. In several viral contexts, AP2-mediated trafficking has been shown to misdirect immune sensors (e.g., MHC-I) away from the plasma membrane while retaining or recycling pro-survival receptors, thereby balancing immune evasion with prolonged host cell viability. By disrupting normal signal transduction, apoptosis, and metabolic pathways, such AP2-mediated manipulation may create a microenvironment conducive to viral persistence.

#### AP2-mediated immune evasion via interaction with antiviral host proteins

AP2 plays a key role in viral immune evasion by counteracting host restriction factors like Tetherin, SERINC, and IFITM3 proteins. These antiviral factors restrict distinct stages of the viral life cycle, and their subversion through AP2-mediated trafficking represents a recurring strategy employed by diverse viruses.

Tetherin, an antiviral protein validated against multiple enveloped viruses, blocks viral budding by inserting its membrane anchor into nascent virions (179). Viral countermeasures against Tetherin vary among lentiviruses: most pandemic-associated HIV-1 group M strains use Vpu to antagonize Tetherin, whereas HIV-1 group O and most SIV strains employ Nef for this function (56). As mentioned earlier, Tetherin is primarily localized within lipid raft microdomains, from which HIV-1 preferentially buds (180). Mechanistically, HIV-1 Vpu contains an EXXXLV motif in its cytoplasmic tail that mediates binding to the AP2S1. Phosphorylation of the serine residues in the Vpu DpS⁵²GxxpS⁵⁶ motif enhances its binding to AP2 (56). Human Tetherin itself possesses a YXXΦ motif that binds AP2M1, facilitating the formation of a Tetherin-AP2-Vpu tripartite complex, which promotes Tetherin internalization and degradation to enable viral immune evasion (57). SIV Nef also contains an EXXXLL motif that mediates interaction with AP2S1 (72). However, unlike related adaptor-binding proteins, Nef has evolutionarily lost the YXXΦ motif and instead acquired a DIWK sequence. Although both Vpu and Nef bind AP2S1, Nef binding induces unique structural changes in the AP2 complex, refolding the first α-helix of the β2 subunit into a β-hairpin that creates a specific binding site for the DIWK sequence (73). This structural remodeling illustrates a distinct mechanism by which SIV Nef hijacks AP2 to counteract host restriction.

SERINC is a family of highly conserved transmembrane proteins with five known members, among which SERINC3 and SERINC5 can inhibit viral infection by suppressing viral membrane fusion (181). HIV-1 Nef utilizes its [E/D]XXXL[L/I] motif to interact with AP2S1 in the unlocked AP2 complex while simultaneously binding SERINC5, thereby forming a SERINC5-Nef-AP2 tripartite complex that is downregulated via the CME pathway. Structural features and other aspects of these interactions have been covered in recent reviews (182). In addition, HIV-1 Nef contains an acidic cluster motif that mediates its interaction with AP2; however, this binding capability does not necessarily extend to AP1 or AP3 (183, 184). Similarly, HIV-2 Nef (185, 186), SIV Nef (74, 186), MLV glycoGag (68, 69), and EIAV S2 (37, 68) exploit nonstructural proteins containing [E/D]XXXL[L/I] motifs to recruit AP2, forming SERINC3- and/or SERINC5-viral nonstructural protein-AP2 complexes that downregulate SERINC3/5 expression and promote immune evasion.

IFITM3 is an interferon-stimulated antiviral protein that inhibits the entry of diverse viruses such as IAV, VSV, and Ebola virus (187). IFITM3 exerts its antiviral activity by modulating membrane structures, disrupting lipid homeostasis, preventing viral fusion, and regulating vesicular pH (188). IFITM3 contains a conserved YXXΦ motif that binds AP2M1, which is important for its antiviral function, as motif mutations impair restriction of above viruses (189). Viruses exploit this interaction by using proteins such as HIV-1 Nef and MLV Gag as molecular scaffolds that simultaneously bind AP2 and IFITM3 to form ternary Nef/Gag-AP2-IFITM3 complexes. This virally facilitated recruitment redirects IFITM3 from the plasma membrane to early endosomes, reducing its incorporation into viral particles and diminishing antiviral activity (190, 191). Collectively, these strategies highlight AP2’s role as a central hub for hijacking antiviral pathways, enabling viral persistence through spatial and functional manipulation of host defenses.

#### AP2-mediated modulation of immune receptor membrane expression for viral immune evasion

AP2 serves as a central hub for viral immune evasion by mediating the downregulation or mislocalization of critical immune receptors. HIV-1 Nef hijacks AP2 to downregulate immune receptors (CD28 [43], CD8αβ [44], CD3 [45], and CD4 [45, 46]), while selectively suppressing mature MHC-II and retaining immature MHC-II on the cell surface to evade T cell recognition (47, 48). Similarly, MCMV employs its m04 protein, which binds AP2M1 via a YRRF motif, to form an m04-AP2-MHC-I complex that accelerates MHC-I internalization, reducing antigen presentation to cytotoxic T cells (27). EBV exploits its viral GPCR BILF1, an oncogenic immune evasion factor, to recruit AP2 and MHC-I to form a BILF1-AP2-MHC-I tripartite complex. This complex redirects MHC-I to lysosomal degradation pathways, effectively crippling adaptive immune responses (20). These examples illustrate how diverse viruses co-opt AP2’s trafficking machinery to disrupt immune receptor surface expression, enabling persistent infection through evading both innate and adaptive immunity.

#### AP2-mediated intracellular transport of viral membrane proteins in immune evasion

AP2 facilitates immune evasion by rapidly internalizing membrane-localized viral proteins from the cell surface, reducing their exposure to immune surveillance and thereby weakening antibody recognition. For instance, the gB protein of PRV hijacks AP2-mediated trafficking to retreat into the cell interior, effectively shielding itself from neutralizing antibodies and evading immune attack (24). This strategy highlights how viruses exploit AP2’s cargo-sorting efficiency to minimize antigenic visibility while maintaining the functional protein dynamics critical for infection.

#### Viral protein-induced AP2 dysfunction facilitates immune evasion

AP2 is exploited by viruses to dysregulate apoptosis and immune surveillance through diverse mechanisms. The E7 protein of HPV competes with EGFR for AP2M1 binding (14), inhibiting EGFR internalization to prolong EGF signaling and drive cell transformation. E7 also modulates the surface expression of MET and CD169 via its interactions with AP2 interactions (15). EBV (20), PLHV (20), and KHSV (23) encode vGPCRs that mimic host GPCRs to induce oncogenic signaling while downregulating MHC-I via AP2 to evade immunity. HAdVs employ the RIDα protein, which contains a YLRH motif (a variant of the YXXΦ motif where histidine acts as a context-dependent hydrophobic residue [7]), to bind AP2M1 and the apoptotic receptor Fas. The resulting Fas-RIDα-AP2 complex internalizes Fas to inhibit apoptosis (8), while parallel AP2-mediated lysosomal degradation of TNFR1 (9) and EGFR downregulation (10, 11) further suppress cell death and promote biosynthesis. Similarly, HIV-1 Nef interacts with AP2A1/2 to manipulate apoptosis—either promoting host cell death to facilitate viral spread or inhibiting it to prolong cell survival, depending on the infection stage (49). These strategies underscore AP2’s central role in viral pathogenesis, balancing immune evasion, apoptosis modulation, and oncogenic transformation to optimize host exploitation.

#### Viral subversion of antiviral signaling pathways via AP2 to evade host immunity

AP2 also intersects with antiviral signaling pathways, such as the TGF-β pathway (192), suggesting that viral proteins exploit their YXXΦ motifs to recruit AP2 via interactions with AP2M1. This recruitment redirects AP2 to manipulate host cell processes, creating a permissive environment for viral replication and spread. Additionally, AP2 interacts with DDX3, a protein involved in tumor progression and immune regulation, to upregulate PD-L1 surface expression, thereby inactivating or eliminating tumor-killing T cells and facilitating immune evasion (193). This AP2-DDX3 partnership highlights a potential mechanism by which oncogenic viruses subvert immune surveillance, linking viral infection to immune escape pathways associated with tumorigenesis.

## ADVANCES IN AP2-TARGETED ANTIVIRAL DRUG DEVELOPMENT

AP2, a critical host trafficking complex, exerts pivotal regulatory roles across multiple stages of viral infection, making it a promising antiviral therapeutic target. Two mechanistic features are particularly critical for AP2 function: (i) phosphorylation of AP2M1 at T156, which enhances AP2 activation and cargo protein engagement, and (ii) recognition of cargo sorting motifs through the hydrophobic binding pocket of AP2M1. Current antiviral drug development focuses on these vulnerabilities. Inhibition of AP2M1 T156 phosphorylation using NAK inhibitors (e.g., sunitinib) blocks AP2 activation and impairs virus-dependent trafficking events. Similarly, small AP2M1 cargo-binding pocket-targeting molecules (e.g., ACA) disrupt AP2-cargo interactions and restrict viral infection. Additionally, experimental inhibition of AP2 expression at the transcriptional or translational level has demonstrated antiviral effects *in vitro* (25, 103), although clinical translation remains challenging due to the essential role of AP2 in cellular homeostasis.

### Advances in antiviral drug development targeting post-translational modifications of AP2

Current drug development targeting AP2 focuses on kinases that phosphorylate the AP2M1 T156 residue, primarily AAK1 and GAK from the NAK kinase family. BM2PK, another member of the NAK family, has also been identified as a potential broad-spectrum antiviral target (194). To date, no studies have reported the involvement of LRRK2 as a kinase phosphorylating AP2 in the context of viral infection processes in host cells.

#### Host-directed broad-spectrum antiviral inhibitors targeting the NAK kinase family

AP2’s phosphorylation, regulated by the NAK kinase family, is a critical target for broad-spectrum antiviral therapy. Sunitinib, a multikinase inhibitor targeting NAK family members (AAK1, GAK, and BMP2K), suppresses AP2M1 phosphorylation and inhibits diverse viruses, including RABV (195, 196), HCV^114^, TOSV (109), Ebola virus (85), DENV (85, 98, 197), ZIKV (85, 198), West Nile virus (WNV) (85), SARS-CoV (199), MERS-CoV (199), SARS-CoV-2 (199), chikungunya virus (CHIKV) (199), Junin virus (JUNV) (199), HIV (199), and respiratory syncytial virus (RSV) (199). Other NAK-targeting agents like erlotinib, 5Z-7-oxozeaenol, gefitinib, and ruxolitinib exhibit similar pan-antiviral potential (Table 2). Notably, the therapeutic feasibility of sunitinib and erlotinib against DENV has been experimentally validated (197), highlighting their translational promise for combating viral infections.

**TABLE 2:** AP2-related antiviral compounds^*a*^

Compound	Status	AP2-related targets	Other targets	Virus
Sunitinib	Approved (cancer)	AAK1 and GAK (AAK1>GAK)	VEGFR2, PDGFRβ, Ire1α, FLT3, KIT, PDGFRα, RET, and CSF-1R	RABV (195, 196)
				HCV (85)
				TOSV (109)
				Ebola virus (85)
				DENV (85, 98, 197)
				ZIKV (85, 198)
				WNV (85)
				SARS-CoV (199)
				MERS-CoV (199)
				SARS-CoV-2 (199)
				CHIKV (199)
				JUNV (199)
				HIV (199)
				RSV (199)
Erlotinib	Approved (cancer)	GAK and AAK1 (GAK>AAK1)	ErbB1, STK10, YSK4, and SLK	SARS-CoV-2 (200)
				DENV (85)
				ZIKV (199)
				WNV (199)
				Ebola virus (85)
				HCV (85)
				CHIKV (199)
				JUNV (199)
				HIV (199)
				RSV (199)
N-(p-amylcinnamoyl) anthranilic acid (ACA)	Experimental	The binding pocket of AP2M1 that competes for cargo binding	PLA2, CaCC, and TRP channel	IAV (87)
				ZIKV (87)
				MERS-CoV (87)
				EV-A71 (87)
5Z-7-oxozeaenol	Experimental	BIKE, GAK, and AAK1	TAK1, VEGFR2, and ERK2	DENV (97)
				Venezuelan equine encephalitis virus (VEEV) (97)
				Ebola virus (97)
Gefitinib	Approved (cancer)	AAK1 and GAK	EGFR	SARS-CoV-2 (201)
				HCV (202)
Ruxolitinib	Approved (myelofibrosis)	AAK1 and GAK	JAK1, JAK2, JAK3, and Tyk2	SARS-CoV-2 (201)
Baricitinib and its derivative	Approved (rheumatoid arthritis)	AAK1	JAK1, JAK2, ROCK1/2, TYK2, CAMK2A, MAP3K2, and PRPF4B	SARS-CoV-2 (201, 203)
1,2,4a,5-tetrahydro-4H-benzo[b] [1,4]oxazino[4,3-d] [1,4]oxazine	Experimental	AAK1	–	SARS-CoV-2 (204)
Tannic acid	Experimental	AAK1	hERG channel and PPARγ	SARS-CoV-2 (205)
Compound 12	Experimental	AAK1	–	SARS-CoV-2 (206)
pyrrolo[2,3-b]pyridine and its derivative (RME-76)	Experimental	AAK1 and BIKE	CDK8, COX-1, COX-2, CDC42BPB, PRKD3, CLK1, CLK4, ULK3, and MINK1	DENV (207–209)
				VEEV (208)
				SARS-CoV-2 (209)
				Ebola virus (209)
Isothiazolo[5,4-b] pyridines	Experimental	GAK	RIPK1	HCV (210)
Isothiazolo[4,3-b]pyridines and its derivative (RMC-242)	Experimental	GAK	–	DENV (209, 211–213)
				HCV (214)
				Ebola virus (194)
				CHIKV (194)
SGC-AAK1-1	Experimental	AAK1 and BMP2K	–	SARS-CoV-2 (215)
SGC-GAK-1	Experimental	GAK	RIPK2, ADCK3, and NLK	SARS-CoV-2 (209)

^*a*^ – indicates that the item has not yet been identified.

AAK1, an upstream kinase of AP2, is a widely studied therapeutic target. It binds directly to the AP2 α subunit and phosphorylates the T156 residue of AP2M1 (216). While dysregulation of AAK1 (e.g., impaired phosphorylation) contributes to bladder cancer (217), studies by Sean D. Conner and Akari Yoshida (218) suggest that AAK1 overexpression neither enhances AP2M1 trafficking nor benefits endosomal transport. Instead, it reduces the number of early endosomes, implying a need for tight stoichiometric control over AAK1-AP2M1 interactions. Clinically, AAK1 inhibitors are approved for the treatment of neuropathic pain by blocking AP2M1 phosphorylation and neurotransmitter trafficking (219). This mechanism is now being repurposed for viral diseases. Notably, the AAK1 inhibitor baricitinib, approved for SARS-CoV-2 clinical trials (220), has demonstrated efficacy in alleviating symptoms and reducing mortality in COVID-19 patients (221), underscoring its therapeutic versatility.

GAK, sharing functional and structural similarities with AAK1, is often co-targeted in antiviral research due to its analogous role in regulating AP2. Like AAK1, GAK binds the AP2 α subunit and phosphorylates AP2M1 (125). Selective isothiazolo-pyridine derivatives (e.g., isothiazolo[4,3-b]pyridine and isothiazolo[5,4-b]pyridine) exhibit high-affinity binding to GAK, specifically inhibiting AP2M1 phosphorylation and suppressing infections by DENV, HCV, Ebola virus, and CHIKV (194, 210–214). These findings position GAK inhibitors as promising broad-spectrum antivirals, mirroring the therapeutic potential of AAK1-targeted agents.

BMP2K, a newly identified NAK kinase family member, also regulates AP2M1 phosphorylation. Both the α and β subunits of AP2 interact with BMP2K in GST pull-down assays (126), although the precise mechanism underlying BMP2K-mediated T156 phosphorylation requires further elucidation. BMP2K inhibitors have demonstrated broad-spectrum antiviral activity, with siRNA knockdown and knockout cell models confirming its critical role in multiple stages of viral infections, including DENV (97, 222). These findings establish BMP2K as a promising antiviral target within the NAK kinase network.

#### Advances in antiviral drug development targeting AP2-binding properties

AP2 promotes viral proliferation through interactions between its cargo-binding subunits (AP2M1 and AP2S1) and viral proteins, highlighting its potential as a therapeutic target. However, drug development aimed at targeting AP2’s binding properties remains in its early stages, with limited studies available to date. One notable report demonstrates that the small-molecule inhibitor ACA blocks AP2M1/YXXΦ interactions, exhibiting broad-spectrum antiviral activity against ZIKV, EV-A71, HIV-1, IAV, adenovirus 5, and MERS-CoV (87). This proof-of-concept underscores the feasibility of disrupting AP2-viral interactions for antiviral therapy, although further mechanistic and translational studies are urgently needed.

### Advances in antiviral strategies targeting AP2 biogenesis

In addition to targeting the AP2 activity, suppression of AP2 expression can also be considered another potential direction for antiviral drug development. Inhibiting AP2 expression at the transcriptional or translational level, for instance, using siRNA or shRNA to deplete specific subunits such as AP2M1, has been proven *in vitro* to impair the replication of diverse viruses, including HIV-1, IAV, and as well as several viruses mentioned above (89, 103). However, it is important to note that these validating studies have so far been conducted primarily in cell culture systems, with progression to *in vivo* models still pending.

This approach directly reduces the cellular pool of AP2 available for co-option by viruses. Although this strategy holds promise as a broad-spectrum intervention, its therapeutic application faces significant challenges, primarily attributable to the indispensable physiological roles of AP2 in cellular homeostasis, which could lead to systemic toxicity and off-target effects (128, 223, 224). Consequently, current drug development efforts are predominantly focused on the more nuanced regulation of AP2’s activity through post-translational modifications. However, direct injection of shRNA targeting AP2A2 or a lipidated AP2 inhibitor peptide into mice effectively alleviated inflammatory pain or postoperative incisional pain (225). Although these studies were not conducted in the context of viral infection, they highlight the feasibility and tolerability of directly targeting AP2 expression or function *in vivo*, demonstrating the great potential of directly targeting AP2 biogenesis or function for antiviral development. The potential and challenges of directly targeting AP2 degradation or expression as a therapeutic strategy are further discussed in the Conclusion and future perspectives section.

## CONCLUSIONS AND FUTURE PERSPECTIVES

AP2 is a pivotal host dependency factor in the life cycles of numerous viruses. From mediating viral entry and regulating genome replication to coordinating virion assembly, release, and immune evasion, the functions of AP2 permeate the entire course of viral infection. AP2 primarily participates in the internalization process of viruses that invade host cells via the CME pathway. For viruses utilizing other pathways, such as members of the Retroviridae family, which mainly invade through membrane fusion (226), AP2 often participates in their post-entry life cycle stages.

During virus-host interactions, viral hijacking of AP2 extends beyond manipulating single pathways. This contains alternative mechanisms, including immune receptor degradation, apoptosis modulation, retention of GPCR, and activation of proliferation-metabolism pathways such as mTOR and Wnt signaling. This reprogramming manifests as a metabolic shift toward substrates required for viral synthesis, suppression of autophagy and other cellular clearance mechanisms, and aberrant enhancement of cell-cycle and proliferation signals, thereby providing the material foundation and spatial-temporal niche for viral replication. Thus, AP2 serves not merely as a “transport coordinator” for virus but also as a central regulatory hub through which viruses remodel the host cellular internal environment. Understanding this system-level manipulation offers critical insights for developing broad-spectrum antiviral strategies that aim to restore cellular homeostasis rather than merely inhibit individual viral proteins.

### Molecular and structural basis of AP2-virus interactions

The current understanding of AP2-virus interactions primarily centers on the specific recognition of YXXΦ and [E/D]XXXL[L/I] motifs in viral proteins by AP2M1 and AP2S1, which enables viruses to hijack AP2-mediated trafficking to complete critical stages of their life cycles. Viruses such as HCV, HIV, and DENV exploit AP2 sequentially in processes including internalization, assembly, and/or release.

However, these motifs are not exclusively specific to AP2; viral proteins can interact with multiple AP complexes to achieve similar outcomes. (i) Different viral proteins interact with other adaptor protein complexes to influence viral processes. For instance, MCMV m04 binds AP2M1 via a YRRF motif, while m154, possibly through a putative AP [DE]D-binding motif, interacts with AP1, and this interaction results in either the accumulation of its target protein in the trans-Golgi network or delivery to lysosomes for degradation (227). (ii) Single viral protein can engage multiple AP complexes to achieve the same function. HIV-1 Vpu, through its EXXXLV motif, is recognized by both AP1 and AP2, potentially reducing Tetherin presence at the plasma membrane via AP1-mediated Golgi trafficking and AP2-mediated endocytic recycling (228). (iii) Distinct viral proteins can bind different AP complexes to orchestrate successive stages of the viral life cycle. Such as HCV, initial cellular entry occurs through the AP2-dependent CME; subsequently, the viral core protein utilizes its YIPL/V motif to bind AP2M1 and coordinate virion assembly; finally, the NS2 protein, potentially via an LXXXD or others sorting motifs, interacts with both AP1 and AP4 to enable mature particle release (95, 229). The broader relationship between other AP complexes and viruses has been reviewed elsewhere (230) and is not elaborated in detail here.

Additionally, certain viral proteins may employ specialized mechanisms to uniquely target specific AP subunits. For instance, serine phosphorylation in HIV-1 Vpu and the presence of an acidic cluster near [E/D]XXXL[L/I] motif in HIV-1 Nef enhance their binding affinity for AP2. Nevertheless, the precise basis for such unique targeting to specific APs requires further investigation. Additionally, SIV Nef can induce a unique conformational change in AP2B1, enabling specific recognition of the DIWK motif in simian Tetherin. These findings suggest that binding of distinct viral proteins may induce specific conformational alterations in AP2, leading to differential trafficking regulation. Collectively, these observations raise a deeper evolutionary question: What selective pressure drives viruses to hijack AP2 rather than other AP complexes? Is it due to the broad subcellular distribution of AP2, or is it the inherent structural and functional plasticity of AP2 that provides a more adaptable platform for viral exploitation? Understanding these molecular and structural principles is critical for the rational design of antiviral strategies targeting AP2-virus interfaces.

### Prospects for AP2-targeted therapeutics

Significant progress has been achieved in the development of AP2-targeted drugs. For example, the YXXΦ motif-binding drug ACA has demonstrated inhibitory effects against multiple viruses *in vivo*, while AP2-associated kinase inhibitors like sunitinib and erlotinib have shown efficacy against Ebola virus and HIV. Ruxolitinib, targeting AP2-related pathways, is currently being evaluated in clinical trials for SARS-CoV-2 (201, 231). Additionally, AP2-virus interactions have enabled the development of innovative drug screening platforms, such as the fluorescence polarization assay developed to identify HIV-1 Nef inhibitors by monitoring CD4 downregulation (232).

As a critical cellular transport factor, AP2 is indispensable for normal cellular life (233), and its dysfunction can lead to a variety of diseases, highlighting the importance of careful evaluation of antiviral drugs targeting AP2 to minimize off-target effects. While kinase inhibitors such as sunitinib exhibit broad-spectrum antiviral activity by targeting AP2-associated kinases (e.g., AAK1/GAK), their clinical translation is limited by side effects including immunosuppression, cardiovascular toxicity, and multikinase inhibitors. Meanwhile, compensatory mechanisms may mitigate these risks. For instance, Huo et al. (96) demonstrated that ZIKV infection elevates AP2B1 levels to facilitate viral entry, yet AP2B1 knockdown alone has no significant impact on ZIKV, suggesting functional redundancy.

Targeted protein degradation (TPD) technology emerges as a compelling future direction for developing therapeutics targeting AP2. Progress and specific details regarding TPD can be found in previously published comprehensive reviews (234). Here, we propose several directions for the development of TPD drugs targeting AP2: (i) PROTACs designed for specific recognition of AP2 subunits with concomitant E3 ubiquitin ligase recruitment to induce proteasomal degradation; (ii) molecular glue degraders capable of inducing neo-interactions between AP2 and cognate E3 ligases; (iii) hydrophobic tagging strategies to trigger quality control-mediated AP2 disposal; and (iv) lysosome-targeting chimeras for shuttling membrane-associated AP2 complexes to lysosomal clearance. For enhanced specificity, conditionally activated degraders represent a compelling alternative, with engineered responsiveness to viral proteases for exclusive AP2 degradation in infected cells, thereby achieving potent antiviral effects while maintaining host cell homeostasis. These findings highlight both the challenges and opportunities in developing AP2-based therapeutic strategies.

### Opportunities for AP2-based vaccines

Viral proteins from multiple distinct virus families contain YXXΦ and/or [E/D]XXXL[L/I] motifs capable of binding to AP2. This functional characteristic could be leveraged for rational vaccine design. Mutating or deleting these interaction motifs in viral proteins may facilitate the development of classical live-attenuated vaccines, mRNA vaccines, nanoparticle-based vaccines, or other platforms.

Strategic modification of viral proteins, either through disrupting existing interaction motifs or introducing exogenous sorting motifs, enables precise control over viral protein trafficking and processing within host cells, thereby allowing rational enhancement of antigen presentation and immune activation. Viral proteins can exploit interactions with AP2 to alter the subcellular localization of immune molecules, cause their aberrant degradation, or trap them within endosomes. This mechanism could potentially be harnessed for recombinant subunit vaccines. For example, introducing YXXΦ motifs and/or [E/D]XXXL[L/I] motifs into proteins containing neutralization epitopes could facilitate AP2-mediated entry into the endosomal pathway, thereby directing these antigens into lysosomes for efficient processing and immune recognition.

Beyond modifying viral antigens, the AP2 complex itself demonstrates a novel target for vaccine adjuvant development. Designing adjuvant molecules with AP2-recognition motifs could actively guide vaccine antigens into the CME pathway, promoting uptake by antigen-presenting cells. Alternatively, directly modulating intracellular AP2 activity could theoretically enhance antigen endocytosis on a global scale, accelerating delivery to processing pathways and ultimately strengthening adaptive immune responses. This strategy provides a new molecular pathway for developing next-generation, high-efficiency vaccine adjuvants.

However, several critical considerations must be addressed: (i) whether the mutated/deleted viral protein maintains its native conformation to remain recognizable by neutralizing antibodies; (ii) whether motif modification successfully attenuates the virus while still stimulating a protective immune response; (iii) whether the modified viral proteins harbor critical neutralization epitopes essential for protective immunity; and (iv) whether the introduction of AP2-targeted adjuvants would produce additional pathological effects. Addressing these challenges will be crucial for successful translation. Overall, the rational manipulation of AP2-virus interactions represents a promising and conceptually innovative avenue for vaccine development, with the potential to simultaneously improve the safety, efficacy, and immunogenicity across next-generation vaccine platforms.
